# Molecular characterization of *Staphylococcus aureus* strains isolated among hospital staff nasal carriers of Babol, Iran

**DOI:** 10.22088/cjim.8.4.311

**Published:** 2017

**Authors:** Parisa Sabbagh, Amirmorteza Ebrahimzadeh-Namvar, Elaheh Ferdosi-Shahandashti, Mostafa Javanian, Soraya Khafri, Mehdi Rajabnia

**Affiliations:** 1 Infectious Diseases and Tropical Medicine Research Center, Health Research Institute, Babol University of Medical Sciences, Babol, Iran.; 2 Department of Microbiology, School of Medicine, Babol University of Medical Sciences, Babol, Iran; 3 Department of Biotechnology, School of Medicine, Babol University of Medical Sciences, Babol, Iran; 4 Department of Statistics and Epidemiology, School of Medicine, Babol University of Medical Sciences, Babol, Iran.

**Keywords:** *Staphylococcus aureus*, PCR, Antibiotic resistance, Molecular characterization, MRSA

## Abstract

**Background:**

*Staphylococcus aureus* (s. aureus) nasal carriers, particularly the healthcare staff can be considered as a potential source for the spread of resistant strains. The aim of this study was to determine the molecular characterization of S. aureus strains isolated among the staff nasal carriers in one of the teaching hospitals in Babol.

**Methods:**

A total of 120 nasal swabs were taken from the staff of Ayatollah Rouhani Hospital Babol during 2016. The antibiotic resistance pattern was performed by disc diffusion method for 13 antibiotics, including cefoxitin, cephalothin, teicoplanin, vancomycin, daptomycin, oxacillin, amoxicillin, amikacin, linezolid, ciprofloxacin, levofloxacin, erythromycin, rifampin, according to the CLSI 2015. Polymerase chain reaction (PCR) was used to detect mecA and pvl genes. Finally, the different SCCmec types were determined by multiplex- PCR method.

**Results:**

Among the 120 collected specimens, 40(33.3%) S. aureus isolates were approved. 28(70%) of strains were identified as methicillin-resistant Staphylococcus aureus (MRSA) and the frequency of pvl gene was confirmed 2(5%). Based on the multiplex PCR assay, four different SCCmec types were detected as 35.7% type I, 14.2% type III, 7.1% type II and 3.5% type IV. By a disc diffusion method, no resistance pattern was observed to vancomycin, while 100% of strains were resistant to amoxicillin.

**Conclusion:**

Consequently our results illustrated that isolated S. aureus strains among the staff nasal carriers via mentioned molecular characterization may lead to increase the nosocomial persistent infections in hospitalized patients and also health care workers.

**A**ccording to the colonization ability of *Staphylococcus aureus* (S. aureus) on the human skin and mucosal membrane, a wide range of community and hospital infections are related to this microorganism (1). Many studies have shown that about 20% of people are known as chronic carriers; however, 30% are regarded as alternate carriers, because the *anterior* nares are recognized as the best ecological niches (endogenous origin). Due to the different studies, S. aureus is accepted as the second leading cause of nosocomial infections (2). Hospital staff nasal carriers are considered as the main source of nosocomial infections, especially in patients undergoing surgery, hemodialysis, cirrhosis, kidney transplant and hospitalized patients (3). Many investigations have proven that the three main reasons are confirming the importance of S. aureus nosocomial and community-related infections, comprised as: I. The rate of S. aureus infections in nasal carriers. II. The existence of various strains with endogenic sources. III. Decreasing the nosocomial infection rate in hemodialysis and patients undergoing surgery with eradication of S. aureus nasal carriers using mupirocin (4). 

In the past decades, penicillin was considered as the first-line treatment, while within a few years by the emergence of beta-lactam resistant strains, the significant global concern occurred. 

The second-generation of semi-synthetic penicillins was introduced in 1959, which inhibits the beta-lactamase enzyme activity; however, the first methicillin-resistant *Staphylococcus aureus* (MRSA) strains were identified in Europe in 1961. Numerous antimicrobial resistance mechanisms were distinguished in S. aureus, for example resistance to methicillin and oxacillin that are encoded by mecA gene. 

This gene is transferred by SCCmec (Staphylococcal cassette chromosome mec), as a mobile genetic element found exclusively in the Staphylococcus genus. Five (I–V) main SCCmec types are classified based on different complex classes and other components. Consequently, hospital acquired-MRSA (HA-MRSA) and community acquired-MRSA (CA-MRSA) are known as an independent deviation. 

Determination of SCCmec various types and also the prevalence of mecA gene can be helpful in monitoring MRSA infections, because the spread of these strains may cause the initiation step of HA-MRSA and CA-MRSA infections (5).

In addition to the variety of virulence factors, surface proteins, and enzymes as well as the rapid development of drug resistance are the prominent potency of this microorganism in clinical infections. For instance, among S. aureus exotoxins, leukotoxin family is able to destroy phagocyte cells such as monocytes and polymorphonuclear (PMN). Leukotoxin consists of two parts: S (slow eluted, 31-32 KD) and F (fast, 36-38 KD) that each part is released separately, but also has synergistic effect on human PMN. Panton-Valentine leukocidin (PVL) is a member of the Staphylococcus leukotoxin family (5) and known as a pore-forming exotoxin. The main role of PVL is initially associated with skin and soft tissue necrosis. Nevertheless, the role of PVL has not been described in serious diseases such as pneumonia. On the other hand, it is not clear why it is often associated with CA-MRSA strains. A study in the Netherlands in 2003 demonstrated that 8% of nosocomial isolates harbored the pvl gene (3). 

The aim of this study was to evaluate the prevalence of S. aureus nasal carriers and determine the different types of SCCmec elements 

## Methods

**Bacterial strains** **: **During a 6-month-period, a total of 120 nasal swabs were collected among the staff of Ayatollah Rouhani Hospital, Babol and transferred to the microbiology lab of Babol University of Medical Sciences for S. aureus isolation and confirmation. Catalase, coagulase, mannitol fermentation and DNase tests were applied.

**Antibiotic resistance assay: **The antibiotic resistance pattern was carried out by a disc diffusion method on Mueller–Hinton agar using cefoxitin (30 µg), cephalothin (30 µg), erythromycin (5 µg), vancomycin (30 µg), linezolid (30 µg), rifampin (5 µg), ciprofloxacin (5 µg), levofloxacin (5 µg), oxacillin (1 µg), amikacin (30 µg), amoxicillin (25 µg) from (Himedia, India) and daptomaycin (30 µg), teicoplanin (30 mg µg) from (Roscoe, Denmark.) according to Clinical and Laboratory Standards Institute (CLSI) 2015. S. aureus ATCC 25923 was used as the control strain (6,7).

**Determination of minimum inhibitory concentration** **(MIC): **E-Test method (Himedia, India) was used for evaluating the vancomycin resistant S. aureus strains. Both S. aureus ATCC29213 and Enterococcus faecalis ATCC 52199 strains were applied as controls.

**DNA extraction: **DNA purification was done using a Roche kit (Roche, Germany) as recommended by the manufacturer. 

**PCR: **All of the strains were determined for the mecA and pvl gene using the PCR test, then were amplified on an A&E thermocyclers (Germany) with the final volume of 20 μl (12.5 µl of Super PCR Master Mix 2X, 3 µl of total DNA, 1 µl of each primer and 2.5 µl of distilled water). Finally, the PCR products were analyzed by electrophoresis on agarose gel (1.5%) with SYBR Safe DNA gel *stain*. Consequently, the PVL positive strains were sequenced (Macrogen, Korea). 

**Multiplex PCR for SCCmec typing: **SCCmec typing was performed for 28 MRSA isolates by a multiplex PCR method in a final volume of 50 µl with MAX PCR Master Mix (Fermentas, Germany) for all PCR reactions (8,9). Specific primers and PCR program are shown in tables1 and 2.

**Table 1:** Specific primers for detection of mecA and pvl genes and SCCmec various types

**Reference**	**Product size(bp)**	**Sequence(5-3)**	**Primer**	**Target**
(7)	533	AAAATCGATGGTAAAGGTTGGC AGTTCTGCAGTACCGGATTTGC	F R	mecA
(8)	433	ATCATTAGGTAAAATGTCTGGACATGATCCA GCATCAACTGTATTGGATAGCAAAAGC	F R	Pvl
(9)	937	ATTGCCTTGATAATAGCCYTCT TAAAGGCATCAATGCACAAACACT	Fb Ra3	Sccmec
	518	CGTCTATTACAAGATGTTAAGGATAAT CCTTTATAGACTGGATTATTCAAAATAT	FccrC RccrC	
	415	GCCACTCATAACATATGGAA CATCCGAGTGAAACCCAAA	F1272 R1272	
	359	TATACCAAACCCGACAACTAC CGGCTACAGTGATAACATCC	F5RmecA R5R431	

* mecA*: methicillin-resistant gene, *pvl*: Panton-Valentine leukocidin, *Sccmec*: Staphylococcal cassette chromosome mec

**Table 2:** PCR programs for detection of mecA and pvl genes and SCCmec various types

**Final extention**	**Extention**	**Annealing**	**Denaturation**	**Initial enzyme activation**	**Cycle**	**Gene **
72 for 5 min	72 for 90 sec	50 for 30 sec	94 for 1 min	94 for 5 min	30	mecA
72 for 5 min	72 for 1 min	55 for 30 sec	94 for 30 sec	95 for 5 min	30	pvl
72 for 4 min	72 for 1 min	55 for 30 sec	94 for 30 sec	94 for 5 min	30	SCCmec

*mecA*: methicillin-resistant gene, *pvl*: Panton-Valentine leukocidin, *Sccmec*: Staphylococcal cassette chromosome mec

## Results

**Figure1:**
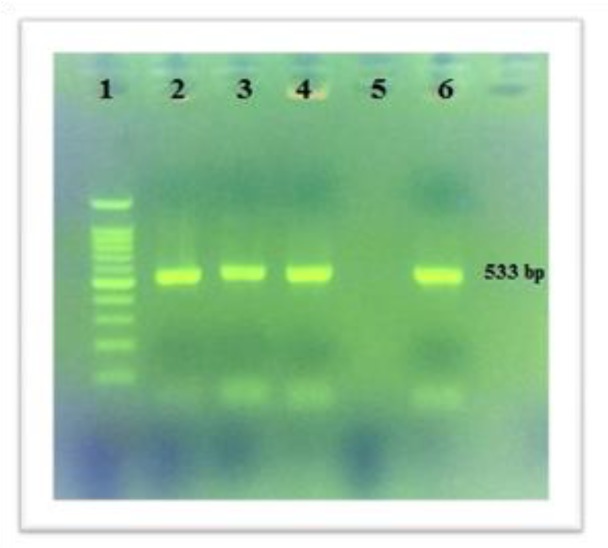
The PCR results of mecA gene for S. aureus strains Lane 1: DNA marker (100bp); Lanes 2, 3 & 4: Positive samples; Lane 5: Negative control; Lane 6: Positive control (ATCC 33591

**Figure2:**
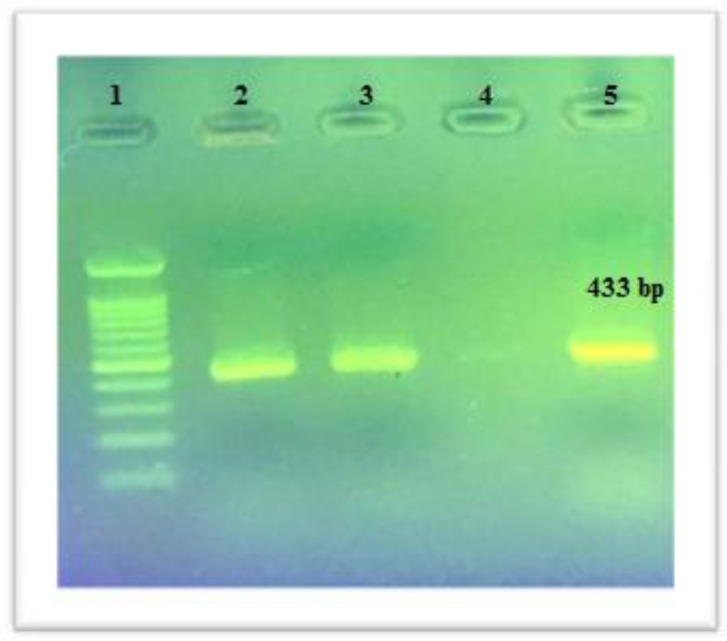
The PCR results of pvl gene for S. aureus strain Lane 1: DNA marker (100bp); Lane 2 & 3: Positive samples; Lane 4: Negative control; Lane: 5 Positive control

**Figure 3:**
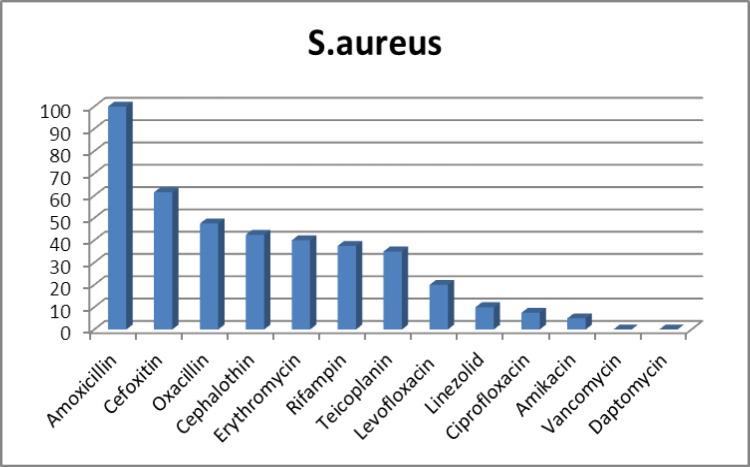
Antibiotic susceptibility pattern of all S. aureus strains

From 120 studied samples, 40 (33.3%) were approved as S. aureus strains. Also, 28(70%) mecA and 2 (5%) pvl genes were distinguished and illustrated in figures 1 and 2. Based on the multiplex PCR assay, four different SCCmec types were detected as follows: 35.7% type I, 14.2% type III, 7.1% type II and 3.5% type IV. In addition, the percentage of HA-MRSA and CA-MRSA isolates were 88.23% and 11.23%. Antibiotic susceptibility test results for the antibiotics are as follows: cefoxitin (61.5%), cephalothin (42.5%), teicoplanin (35%), vancomycin (0%), daptomaycin (0%), oxacillin (47.5%), amoxicillin (100%), amikacin (5%), linezolid (10%), ciprofloxacin (5%), levofloxacin (20%), erythromycin (40%) and rifampin (37.5%) (Figure 3). It is noteworthy that there was no any resistance pattern to vancomycin in E-test method (MIC≤ 2 μg/mL).Statistical data in accordance with nasal carriers in health care different units have been demonstrated in details (Table 3). In this study, the percentage of HA-MRSA isolates had the highest rate in comparison to CA-MRSA, likewise, the highest percentage (80%) of MRSA isolates belonged to the nurses .

**Table 3:** S. aureus strains isolated from different hospital wards

**wards**	**Staphylococcus aureus frequency(%)**
Laboratory	27/3
Maternity ward	33/3
ENT	28/6
Orthopaedics	28/6
Cardiology	22/2
Gastroenterology	30
Neurology	50
Endocrinology	0
ICU	42/9
Operating room	36/4
Emergency	33/3
haematology	28/6
Infectious disease	63/6
NICU	50

## Discussion

In the past few decades, S. aureus is considered as one of the most common nosocomial infections due to various virulence factors, acquisition of antibiotic resistance genes, the poor *sanitation* and hygiene of health care setting and discriminate use of antibiotics. However, hospital staff nasal carriers can be effective in this issue (10)**. **In our study, the prevalence of S. aureus strains among hospital staff was found 33.3% similar to other research reports which were conducted by Lamaro-Cardoso et al. in Brazil and Davoodabadi et al. in Isfahan (11,12). Moreover, in related studies, the rate of nasal carriers reported 38.5% and 28.9%, respectively (13,14). In general, the incidence of S. aureus nasal carriers is estimated between 10% and 70% worldwide (15). Yet different risk factors like gender, age, alcohol consumption, smoking, chronic diseases (e.g. diabetes and skin diseases) may affect the mentioned ratio (16). The lowest ratio of nasal carriers in a certain unit (endocrinology) may indicate the heeding of the principles of health and sanitation rules in comparison to infectious diseases and NICU wards, albeit this prominent percentage in infectious diseases ward is not unexpected. It should be mentioned that there was no coloration between nasal carriers and age, sex, education and occupation (P< 0.05), whereas, Diawara et al. and Gebreyesus et al. have shown the relation between age and gender with nasal carriers (17,18).

In the present study, all of the strains were susceptible to vancomycin, and resistant to amoxicillin, this reflects the fact that penicillin is not very effective in the treatment. On the contrary; vancomycin still acts as a therapeutic agent against S. aureus strains. The results of this study presented that 87.5% of the strains were resistant to more than two antibiotics, so due to the spread of MDR strains, this may become a considerable challenge for treatment process. Additionally, 10% of the strains were resistant to linezolid, while there were no reports of any resistance to this antibiotic in Delost et al.’s study (19)**. **From 1968 to 2003, only two new antibiotic classes (linezolid and daptomycin) were proposed for the treatment of S. aureus infections by the FDA, some case reports of antibiotic resistance were observed (20,21). 

The frequency of mecA gene using the PCR method was 70%, though via the disc diffusion was 47.5%. In the related study by Namvar et al. in 2014 among 40 S. aureus clinical isolates, 75% were resistant to oxacillin and 100% of them harbored mecA gene (22). Molecular typing is necessary to determine the origins of the bacteria strains, clonal relations and epidemiological research studies play an important role in the prevention and treatment of infections. Several phenotypic and genotypic methods could be applied for classifying strains used in epidemiological studies, detection and investigation of hospital infection outbreaks (23). 

Classification and identification of MRSA strains require a thorough understanding of the genetic structure and detection of SCCmec types. In this study, the SCCmec typing for 28 MRSA strains were 35.7% type I, 14.2% type III, 7.1% type II and 3.5% type IV. These results indicated that the high rate of our studies isolated belonged to HA-MRSA. Jina Lee in a study which was carried out in 2010 in Korea showed that the main SCCmec was type 4, while type 2 had the lowest prevalence (24). Huang studies in Taiwan reported only two types of SCCmec (types IV & V) (25), whereas, in Namvar et al.’s study, the clinical samples demonstrated that the most type of SCCmec was related to type III (47.5%) and the lowest prevalence was type I (7.5%), thus, 65% and 35% of strains were HA-MRSA and CA-MRSA, respectively (22). According to the studies conducted, it was predicted that CA-MRSA strains are common amongst nasal carriers, nevertheless, our results were similar to clinical samples outcome. These results indicate warning signal since the hospital staff is in close contact with patients, the probability of S. aureus transmission increased.

According to results, the highest percentage (80%) of MRSA isolates belonged to the nurses, which was similar to other studies (18, 26) that this fact can be interpreted by the high contact between nurses with patients and other health care workers. On the other hand, the pathogenicity of MRSA strains depends on virulence factors such as PVL toxin (27) that can cause apoptosis, necrosis and may accelerate the destruction of the polymorphonuclear and mononuclear human cells (4). In this study, the pvl gene frequency was 5%, nonetheless in related studies were 19% and 4.8%, respectively (13,28). Two positive pvl strains were sequenced and established in NCBI database (Accession no: KY322721 & KY322722). The relation between S. aureus nasal carriers and nosocomial infections has been demonstrated (29), consequently, the presence of pvl positive carriers has a particular importance (5). As a result, the high rates of resistance to various antibiotic classes is a critical concern, accordingly, an antimicrobial surveillance program to promote the proper use of antibiotics is required, and besides our results illustrated that S. aureus strains isolated among the nasal carriers with mentioned molecular characterization may lead to the increase of nosocomial persistent infections in hospitalized patients and hospital staff.
